# Influence of Optical Brightening Agent Concentration on Properties of Cotton Fabric Coated with Photochromic Microcapsules Using a Pad-Dry-Cure Process

**DOI:** 10.3390/polym11121919

**Published:** 2019-11-21

**Authors:** Mateja Kert, Vida Krkoč, Marija Gorjanc

**Affiliations:** 1 Department of Textiles, Graphic Arts and Design, Faculty of Natural Sciences and Engineering, University of Ljubljana, 1000 Ljubljana, Slovenia; marija.gorjanc@ntf.uni-lj.si; 2 Tekstina tekstilna industrija d.o.o., 5270 Ajdovščina, Slovenia; vida.krkoc@tekstina.si

**Keywords:** photochromic microcapsules, photocoloration, cotton, optical brightening agent, pad-dry-cure process

## Abstract

The weak photostability of photochromic dyes applied to textile substrates is one of the disadvantages of the broader use of photochromic dyes in the textile industry. Therefore, the influence of optical brightener concentration on both the photocoloration and photostability of cotton fabric coated with photochromic microcapsules using a pad-dry-cure process, as well as the physical-mechanical properties and colorfastness properties, were studied in this research. Coated samples were subjected to different tests according to valid EN ISO standards; namely mass per unit area, fabric stiffness, breaking force and elongation, air permeability, and different colorfastness properties (rubbing, domestic and commercial laundering, and light). Results showed that the coated fabric had higher mass per unit area, stiffness, breaking force and elongation and lower air permeability compared to uncoated fabric, irrespective of the padding bath composition. Coated fabric has better colorfastness to wet than dry rubbing. The colorfastness to washing decreases with the increased number of washing cycles. The use of optical brightener decreases the photocoloration of coated fabric and increases the photostability of coated fabric after the exposure of samples to a Xenotest apparatus for longer than 12 h.

## 1. Introduction

Optical brighteners are generally used to raise the whiteness of chemically bleached textile substrates. They are fluorescent chemicals that absorb in the ultraviolet (UV) region of the spectrum and emit light in the blue-violet region of the visible spectrum. The most common optical brighteners for cellulosic fibers are triazinyl derivatives of diaminostilbenedisulphonic acid with different substituents, on which substantivity and application method depends [1]. The optical brightener itself cannot change any physical-mechanical properties of fabric after application, but its working mechanism could have an influence on photochromic characteristics of photochromic dyes.

Photochromic organic compounds have an ability to transform from form A to form B and vice versa. The reversible transformation is induced in one or both directions by electromagnetic radiation between two stages possessing observable light absorptions in different regions [2]. Photochromic dyes can be applied onto textiles by exhaust dyeing, solvent-based dyeing methods, printing, or using sol-gel technology [3,4,5,6,7,8,9,10,11,12,13,14,15,16,17,18]. On glass plates, they are usually applied by sol-gel technology [19,20,21]. The incorporation of photochromic dye into the fiber is also established [22,23]. In general, photochromic dyes are poorly exhausted on textile substrates [4,6] due to their highly non-planar ring-closed form and the deficiency of substituents, which increases the solubility of the dye in an aqueous medium. Consequently, the poor photochromic color build-up is obtained on textile substrates. Furthermore, the study of the synthesis and application of water-soluble photochromic dyes, and the study of photochromic dye with a reactive anchor are already published [5,8]. Commercial photochromic dyes are often in the form of microcapsules [3], and thus they are applied onto textile substrates by pad-dry-cure processes, coating, or printing [24,25,26] and the photochromic color build-up is more pronounced. Moreover, plasma treatment could even increase the embedment of UV-responsive microcapsules to polyethylene terephthalate fabric during the pad-dry-cure process [27].

In ophthalmics, photochromic dyes achieved commercial success, but in the textile industry, they are rarely represented due to poor colorfastness to light and washing [3]. The other reason could lie in the non-conventional dyeing process for their application to textiles, due to the form in which commercial photochromic colorants are supplied [3]. Research carried out so far showed weak photostability of photochromic dyes applied to textiles, which can be improved by the use of UV-absorbers or hindered amine light stabilizers (HALS) [15]. From the study, researchers established that the photostability is strongly dependent on the concentration and structure of the photochromic dye, UV-absorbers, and HALS. The light fatigue resistance of spiropyranes can be also improved by the addition of nickel or zinc 4,8-dihydroxyquionline-2-carboxilated, which causes the stabilization of the dye due to the contribution of bifunctional amphoteric counterions [28]. Furthermore, studies on the influence of optical brightener concentration on the photocoloration and photostability of microencapsulated photochromic dye applied onto the cotton fabric by pad-dry-cure processes were not found. Therefore, the aim of our research was to establish if the addition of an optical brightener into the padding bath could reduce the background color and improve the photostability of the fabric coated with photoresponsive microcapsules. Thus, four different concentrations of optical brightener were used in the research. It was assumed that the addition of the optical brightener will influence both the intensity of the developed color after exposure of the coated fabric to ultraviolet A (UVA) light and the photostability of the coated fabric. Moreover, physical-mechanical characteristics of coated fabrics were also studied.

## 2. Materials and Methods

### 2.1. Fabric

Desized, scoured and chemically bleached 100% cotton fabric from the producer Tekstina Ajdovščina (plain weave, mass of 119.79 g/m^2^, warp density 50 threads/cm, weft density 30 threads/cm) was used in the research.

### 2.2. Chemicals

Commercial microencapsulated photochromic dye (Itofinish UV blue, MagnaColours, Barnsley, UK), acrylic copolymer (Itobinder AG, MagnaColours, Barnsley, UK) and optical brightener (Ultraphor CK, BASF, Ludwigshafen, Germany), a derivate of stylbenedisulphonic acid, were used in the research.

### 2.3. Textile Coating with Photochromic Microcapsules

Photoresponsive microcapsules were applied onto the cotton fabric by a pad-dry-cure process. The composition of the padding baths is presented in Table 1. The wet pick-up was 98%. After padding, the cotton fabric was dried for 2 min at 100 °C and then cured for 4 min at 150 °C.

### 2.4. Methods

#### 2.4.1. Mass per Unit Area

The mass per unit area, expressed in g/m^2^, of the studied samples was determined using the EN 12127: 1999 standard.

#### 2.4.2. Stiffness

The stiffness of uncoated and coated samples was determined using method A of the ASTM D1388-64 standard. Stiffness in the warp and weft directions, expressed in mg·cm, was calculated from Equation (1). Overall flexural rigidity (*U_k_*) was expressed from Equation (2).

(1) $$U_{o,v}=\;T\cdot {\left(\frac{{\bar{l}}_{o,v}}{2}\right)}^{3}$$

(2) $$U_{k}=\;\sqrt{U_{o}\cdot U_{v}}$$

where T is the mass per unit area in g/m^2^, and $\bar{l}$ is an average value of bending length in warp (*o*) or weft (*v*) direction in cm.

#### 2.4.3. Breaking Force and Elongation

The tensile rigidity of the fabric was determined using the EN ISO 13934-1: 1994 standard. Some modification of the standard was performed. Namely, from each studied sample, five test pieces in the warp and five test pieces in the weft direction were cut out, with dimensions of 150 mm × 25 mm. Each test piece was subjected to tensile loading by an Instron 5567 dynamometer (Instron, Buckinghamshire, UK) with a fasten length of 50 mm and a velocity load of 100 mm/min.

#### 2.4.4. Air Permeability of the Textile

The air permeability of the uncoated and coated samples was analyzed by an Air-Tronic 3240B apparatus (Mesdan, Brescia, Italy), using the ISO 9237: 1995 standard. On each sample, 10 measurements were done at a pressure of 500 ± 2 Pa, with a testing area of 100 cm^2^.

#### 2.4.5. Spectrophotometric Measurements

The color of coated samples was analyzed using a Datacolor SF 600 PLUS-CT spectrophotometer (Lawrenceville, NJ, USA). Reflectance (*R*) and CIELAB color coordinates were determined before and after the irradiation of coated samples to UVA light for 1 min using two Philips TL-D 18W actinic bulbs. The distance between the bulbs and the sample was 11 cm. All measurements were performed using four layers of fabric with a 9 mm aperture, with specular reflectance included and the UV component excluded (0% UV, filter FL40 on), under D_65_ illumination and with a 10° standard observer. An average of five measurements were taken for each sample. Due to dynamic color change, which is specific for photochromic dyes, the colorfastness properties of coated samples were evaluated from the values of color difference (ΔE*_ab_) [13,15], calculated between tested (colorfastness to rubbing, laundering and light) samples before and after irradiation with UVA light for 1 min and compared to those before testing.

#### 2.4.6. Colorfastness to Rubbing

The testing of the colorfastness of coated samples to rubbing was performed on a Crockmeter M238BB (SDL ATLAS, Rock Hill, SC, USA) using the EN ISO 105-X12: 2002 standard.

#### 2.4.7. Colorfastness to Domestic and Commercial Laundering

The colorfastness to domestic and commercial laundering was performed according to the EN ISO 105-C06: 2012 standard. Washing was performed in a GyroWash apparatus (James Heal, Halifax, UK) using test methods A1S and A1M. European Colourfastness Establishment (ECE) detergent without a fluorescent whitening agent was used as a washing agent. According to the standard, the results of one multiple (M) test may in some cases be approximated by the results of up to five domestic or commercial launderings at temperatures not exceeding 70 °C. Thus, the test method A1M was performed twice, corresponding to 10 washing cycles.

#### 2.4.8. Colorfastness to Light

Studied samples were exposed to a xenon arc lamp for 1, 6, 12, and 24 h in a Xenotest Alpha apparatus (Atlas, Rancho Cucamonga, CA, USA). Samples were prepared according to EN ISO 105-B02:2014 standard and subjected to testing.

#### 2.4.9. Scanning Electron Microscopy (SEM)

SEM JSM-6060LV (JEOL, Tokyo, Japan) was used to take SEM micrographs of S2 and S3_4 samples before and after dry and wet rubbing at 200× magnification. The average diameter of microcapsules was 5.59 μm, with a standard deviation of 1.60 μm. Measurements of microcapsule diameter, taken from SEM micrographs at 700× magnification, were determined on 50 microcapsules.

## 3. Results and Discussion

### 3.1. Physical-Mechanical Properties of Samples

#### 3.1.1. Mass per Unit Area

**Figure 1:**
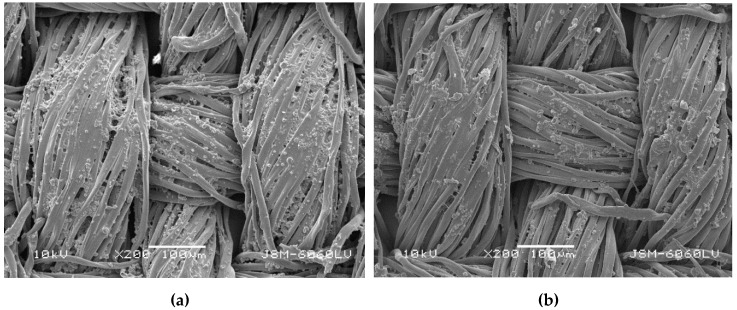
SEM (scanning electron microscope) micrographs of samples S2 (**a**) and S3_4 (**b**) at 200× magnification.

The coated samples had a higher mass per unit area than uncoated ones (Table 2), which is expected. From the values of *T,* (samples S3_1–S3_4) it can be seen that the concentration of optical brightener (OB) influences the values of *T*. With the increase in the concentration of OB in the padding bath (samples S3_1–S3_4), a decrease in *T* is noticed. One of the possible explanations could be the increase in the negative charge on fiber surface due to the adsorption of OB, containing SO_3_^-^ groups that can repulse the application of the anionic binder into which photochromic microcapsules are entrapped. This was confirmed by SEM micrographs of samples S2 and S3_4 (Figure 1). They showed that sample S3_4, whose padding bath includes the highest amount of OB, contains a lower number of microcapsules compared to sample S2, which was padded without OB.

#### 3.1.2. Stiffness

All samples had higher flexural rigidity in the warp than in the weft direction (Table 2), which is in accordance with the constructional parameters of the fabric. The fabric has a higher density in the warp than in the weft direction. Coated samples had higher stiffness in both directions warp and weft than the uncoated samples, which is in line with our expectations. During the curing process, the binder forms a binder layer between cotton fibers into which microcapsules are entrapped, which could cause the increase of fabric stiffness. Even though the values of *U_K_* differ among samples S3, the fluctuation of values could be ascribed to binder layer and entrapped microcapsules rather than the concentration of OB.

#### 3.1.3. Breaking Force and Elongation

From the results, collected in Table 2, it can be seen that the breaking force (*F_p_*_r_) of the fabric increases after the pad-dry-cure process. One of the possible reasons could be that the binder forms a binder layer during the curing process and thus fills the voids between fibers. Fibers within yarns become stronger and therefore a higher force is needed at the break. On the other hand, no deterioration of elongation at break (*ɛ_pr_*) is noticed for coated samples, and it even improves slightly. A possible explanation for such a phenomenon could be that the binder layer slightly increases the elasticity of the coated fabric. Moreover, it should be stressed that the size of the test piece was 150 × 25 mm and the fasten length was 50 mm, which could also contribute to the obtained results. With the increase in OB concentration in the padding bath, no proportional increase or decrease in the values of *F_pr_* and *ɛ_pr_* is noticed, which clearly shows that the OB has no influence on breaking force and elongation of coated fabric.

#### 3.1.4. Air Permeability

Results in Table 3 show that the uncoated sample (S1) has essentially higher air permeability than coated samples. The lowest air permeability is shown by sample S2, padded in a bath containing photoresponsive microcapsules and binder, whilst the highest air permeability is noticed in sample S5, padded with binder only. The latter result suggests that the binder does not form a continuous layer on the fabric surface after curing and also does not close the pores between the warp and weft threads, and therefore the fabric remains sufficiently permeable for air. On the other hand, microcapsules can form a uniform, thin and continuous coating on the fabric surface and thus fix the spaces in the fibers [25]. The reduction of air permeability of sample S2 is therefore attributed to the synergistic effect of both ingredients in the padding bath, i.e., binder and microcapsules. It can also be seen from Table 3 that the air permeability of the fabric increases with the increase in OB concentration in the padding bath. This is additional evidence that the addition of OB into the padding bath reduces the adhesion between fiber and binder, and therefore a lower number of microcapsules is entrapped into the cured binder layer. The latter result is shown by SEM micrographs of samples S2 and S3_4, presented in Figure 1.

### 3.2. Reflectance, Color and Colorfastness Properties of Tested Samples

#### 3.2.1. Reflectance

**Figure 2:**
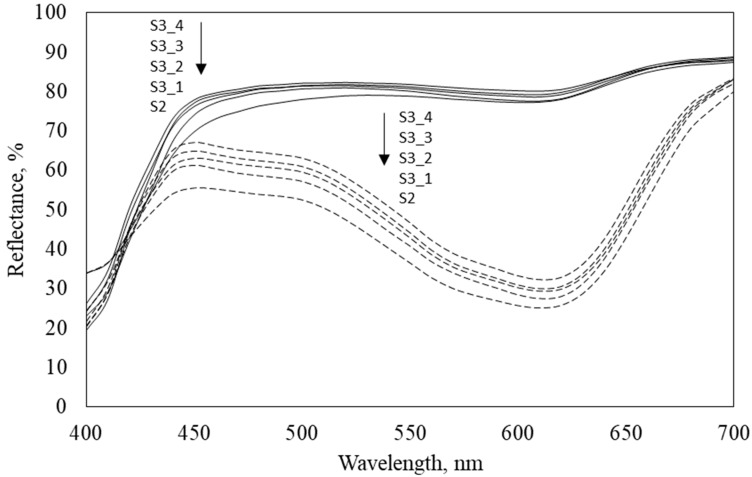
Reflectance curves of coated samples before (—) and after (‒ ‒) irradiation with UVA light.

From reflectance curves of coated samples before irradiation with UVA light, presented in Figure 2, it can be seen that the addition of OB (samples S3_1–S3_4) increases reflectance values of coated samples in the studied range of wavelengths in comparison with sample S2. The reflectance slightly increases by the increase of OB concentration. After irradiation of the coated samples with UVA light, the decrease of reflectance is noticed, irrespective of the addition of OB, due to the transformation of photochromic dye from colorless to colored form under UVA light. The influence of OB concentration on reflectance values is more pronounced for irradiated samples. The reflectance increases by the increase in OB concentration in the padding bath since OB and photochromic dye absorb UV light simultaneously. The wavelength of absorption maximum (λ_max_) of studied encapsulated photochromic dye on cotton fabric is 610 nm. The addition of OB has no influence on λ_max_ of the studied dye.

#### 3.2.2. Color of Tested Samples

From CIELAB values of uncoated and coated cotton fabric (Table 4), it can be seen that the coated cotton fabric becomes darker (value of CIE L* decreases), greener (value of CIE a* decreases), and yellower (value of CIE b* increases) after the application of photochromic microcapsules. Moreover, the application of photochromic microcapsules affects the background color of the coated cotton fabric. When OB was added into the padding bath, the cotton fabric became slightly lighter, less green, and less blue. After the exposure of the coated fabric to UVA light for 1 min (Table 5), the fabric becomes even darker, greener (value of CIE a* decreases), and bluer (value of CIE b* decreases). With the increase in OB concentration in the padding bath, samples from S3_1 to S3_4 become lighter and less blue, whilst its influence on the value of CIE a* is less perceptible.

#### 3.2.3. Colorfastness to Rubbing

**Figure 3:**
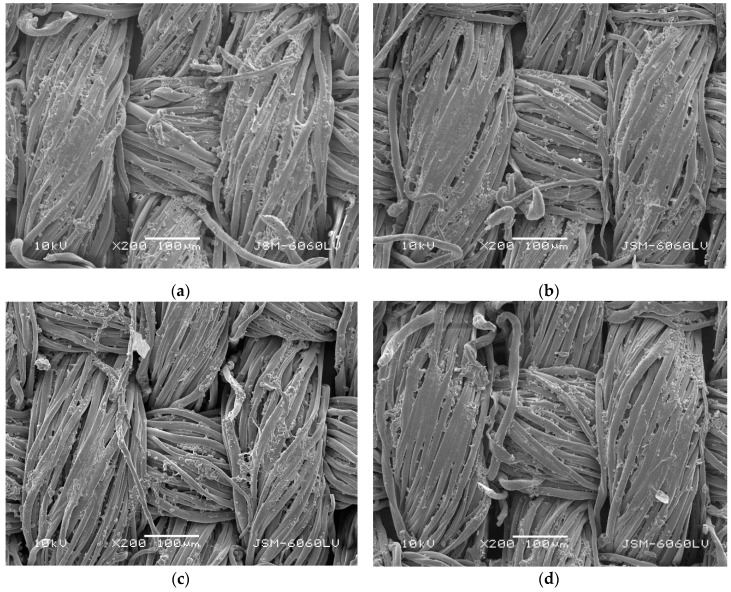
Sample S2 after wet (**a**) and dry (**b**) rubbing and sample S3_4 after wet (**c**) and dry (**d**) rubbing at 200× magnification.

The values of ΔE*_ab_ in Table 6 show that rubbed samples had lower ΔE*_ab_ than unrubbed samples. The decrease in ΔE*_ab_ is a consequence of the removal of the cured binder layer into which microcapsules are entrapped. Since the used microcapsules had no affinity to cellulosic fiber, the adhesion between microcapsules and fibers is dependent upon the binder, present in the padding bath. It can be concluded that only microcapsules were removed during rubbing, which were more present on the fabric surface than microcapsules entrapped in the cured binder layer among threads of fabric. The values of ΔE*_ab_ show that the colorfastness of coated samples to rubbing is slightly influenced by the type of rubbing. With wet rubbing, higher values of ΔE*_ab_ were obtained compared to dry rubbing. The cause for the removal of the cured binder layer into which microcapsules were entrapped at dry rubbing was ascribed to higher friction between the coated fabric and dry cotton fabric in comparison to wet rubbing, where coated fabric is rubbed with wet cotton fabric [26], which was confirmed by SEM micrographs of samples S2 and S3_4 (Figure 3a–d), showing that the cured binder layer into which microcapsules were entrapped was removed to a greater extent from the upper than from the lower threads and from the surface, which was more exposed to rubbing. The same observation was noticed in our previous study [26]. The lower values of ΔE*_ab_ of unrubbed samples S3_1–S3_4 compared to unrubbed sample S2, were ascribed to a lower application of binder into which microcapsules were entrapped due to the presence of OB in the padding bath on one hand, whilst on the other hand the optical brightener and the photochromic dye absorb UV-light at the same time, resulting in a lower photocoloration of the fabric.

#### 3.3.4. Colorfastness to Domestic and Commercial Laundering

The values of ΔE*_ab_ of washed samples decreased compared to the ΔE*_ab_ of unwashed samples (Table 7), meaning that the microcapsules embedded into the binder layer were removed from the cotton fabric after the first washing cycle, whilst after 10 washing cycles an even higher decrease was noticed. It was concluded that only microcapsules that were not strongly embedded into the cured binder layer were removed during the laundering process. The results of ΔE*_ab_ of samples S2 and S4 clearly show that the binder increases the adhesion between microcapsules and fiber, and therefore a higher colorfastness to laundering of sample S2 is achieved compared to sample S4. The drop in the values of ΔE*_ab_ after the first and the tenth washing cycles of sample S4 is higher compared to sample S2. The lower values of ΔE*_ab_ of sample S4 compared to sample S2 show that a lower number of microcapsules was coated onto the cotton fabric in the case of sample S4 than sample S2. The latter result is in accordance with our expectations since padding bath B6 did not include a binder as in the case of padding bath B1 (sample S2). For unwashed samples, the decrease in the value of ΔE*_ab_ with the increase in OB concentration in the padding bath was noticed, as a consequence of lower photocoloration of samples on one hand and a lower amount of microcapsules coated on the cotton fabric on the other hand due to repulsive forces between adsorbed OB and the anionic binder.

#### 3.3.5. Colorfastness to Light

**Figure 4:**
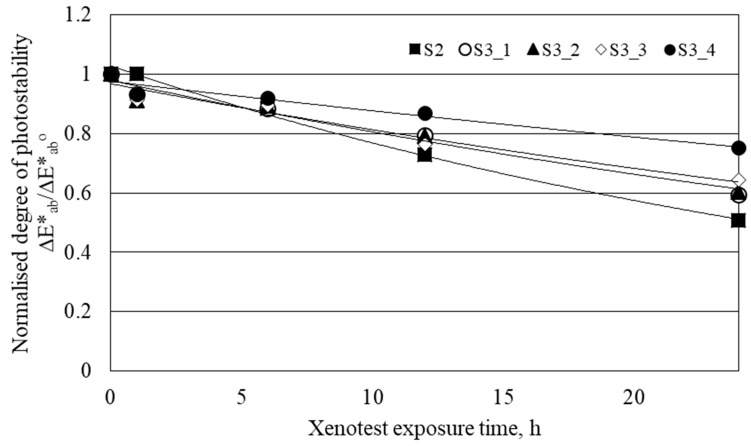
Normalized degree of photostability of studied samples.

After the illumination of samples in the Xenotest apparatus, the values of ΔE*_ab_ of the studied samples decreased (Table 8). It is known that UV-absorbers increase the colorfastness to light of photochromic textiles [15]. Since the optical brightener absorbs UV-light, it was assumed that the addition of an optical brightener into the padding bath will improve the colorfastness to light of photochromic textiles. The latter was confirmed with the normalized degree of photostability [15] of the studied samples (Figure 4), which clearly shows that after illumination longer than 12 h, samples from S3_1 to S3_4 have higher photostability (higher values of ΔE*_ab_/ΔE*_ab_^o^) than sample S2. According to the obtained results, it can be concluded that the colorfastness of microencapsulated photochromic dye to light, applied to cotton fabric, could be improved even at low concentrations of OB. At the same time, a slight change in background color was noticed on fabric after the application of microcapsules of photochromic dye and OB.

## 4. Conclusions

In this research, desized, scoured and chemically bleached cotton fabric was successfully coated with microcapsules of photochromic dye in the absence and the presence of optical brightener of different concentrations using a pad-dry-cure process. The increase of optical brightener concentration in the padding bath decreases the application of binder, into which microcapsules of photochromic dye are entrapped. After coating the flexural rigidity of the fabric increased in the warp and weft directions, irrespective of the absence or the presence of an optical brightener in the padding bath. The breaking force increased, whilst elongation of the coated fabric slightly improved. The air permeability of the coated fabric decreased, albeit to a smaller extent, when an optical brightener was present in the padding bath. The coated fabric had better colorfastness to wet than to dry rubbing, whilst the colorfastness to washing decreased with the increasing number of washing cycles. The presence of optical brightener and its concentration in the padding bath has no influence on colorfastness of coated fabric to rubbing and washing whilst its influence on light fastness is more pronounced. The colorfastness to light of the coated fabric improved with the increased concentration of optical brightener. In order to determine whether the optical brightener could be a good substitute for UV-absorbers or HALS, further research should be performed to confirm or deny this claim.

## Figures and Tables

**Table 1:** Composition of the padding bath and sample labeling.

Sample	Padding Bath	Concentration (g/L)		
		Itofinish UV Blue	Itobinder AG	Ultraphor CK
S1	-	-	-	-
S2	B1	100	50	0
S3_1	B2	100	50	0.2
S3_2	B3	100	50	0.4
S3_3	B4	100	50	0.6
S3_4	B5	100	50	0.8
S4	B6	100	0	0
S5	B7	0	50	0

**Table 2:** Mass per unit area (*T*), stiffness in the warp (*U_o_*) and weft (*U_v_*) directions, overall flexural rigidity (*U_k_*), breaking force (*F_pr_*) and breaking elongation (*ɛ_pr_*) in the warp and weft directions of the studied samples.

Sample	*T* [g/m^2^]	*U_o_* [mg·cm]	*U_v_* [mg·cm]	*U_k_* [mg·cm]	*F_pr_* [N]		*ɛ_pr_* [%]	
					Warp	Weft	Warp	Weft
S1	119.79	307.53	110.94	184.71	302.44	216.10	13.23	16.70
S2	131.49	430.54	205.30	297.30	380.31	235.46	19.33	17.47
S3_1	131.78	431.82	171.02	271.75	326.29	226.41	18.60	20.93
S3_2	131.63	451.86	218.26	314.04	371.46	224.18	19.83	15.97
S3_3	129.16	443.38	178.55	281.36	342.36	212.24	17.80	18.60
S3_4	127.94	439.19	188.15	287.46	357.23	244.33	18.87	18.00

**Table 3:** Average values of airflow through the fabric ($\bar{q}$) and air permeability (*R*) of the studied samples.

Sample	$\bar{q}\;[{\mathrm{dm}}^{3}/\min]$	*R* [mm/s]
S1	91.0	303.8
S2	53.1	177.3
S3_1	53.6	179.0
S3_2	53.7	179.2
S3_3	68.3	228.1
S3_4	71.8	239.7
S4	52.6	175.7
S5	78.2	261.2

**Table 4:** The CIELAB color values, chroma (C*_ab_) and hue (h_ab_) of uncoated and coated samples before exposure to UVA light.

Sample	L*	a*	b*	C*_ab_	h_ab_ (°)
S1	94.21	−0.33	3.17	3.19	95.96
S2	91.60	−5.24	8.37	9.87	122.08
S3_1	91.96	−5.43	7.64	9.37	125.40
S3_2	91.83	−5.26	7.16	8.89	126.31
S3_3	92.26	−4.81	6.40	8.01	126.89
S3_4	92.24	−4.97	6.63	8.29	126.85

**Table 5:** The CIELAB color values, chroma (C*_ab_) and hue (h_ab_) of coated samples after exposure for 1 min to UVA light.

Sample	L*	a*	b*	C*_ab_	h_ab_ (°)
S2	71.41	−11.76	−15.31	19.31	232.49
S3_1	72.73	−12.63	−15.32	19.86	230.47
S4_2	74.33	−11.93	−14.25	18.59	230.05
S5_3	76.13	−11.82	−13.07	17.62	227.84
S3_4	77.28	−11.90	−12.39	17.19	226.13

**Table 6:** Colorfastness to rubbing of studied samples.

Sample	ΔE*_ab_		
	Without Rubbing	Wet Rubbing	Dry Rubbing
S2	31.79	31.55	31.22
S3_1	30.80	28.69	27.88
S3_2	28.44	24.69	24.40
S3_3	26.24	24.51	23.38
S3_4	25.18	22.98	21.98

**Table 7:** Colorfastness to domestic and commercial laundering.

Sample	ΔE*_ab_		
	Unwashed	After One Washing Cycle	After Ten Washing Cycles
S2	31.79	30.96	28.50
S3_1	30.80	29.58	26.96
S3_2	28.44	27.35	25.82
S3_3	26.24	25.31	24.55
S3_4	25.18	24.64	23.48
S4	26.65	24.41	16.40

**Table 8:** Color difference before (ΔE*_ab_^o^) and after different hours (ΔE*_ab_) of illumination.

Sample	ΔE*_ab_^o^	ΔE*_ab_			
	0 h	1 h	6 h	12 h	24 h
S2	31.79	31.85	28.15	23.20	16.10
S3_1	30.80	28.66	27.19	24.49	18.32
S3_2	28.44	25.87	25.39	22.50	17.18
S3_3	26.24	23.93	23.50	20.00	16.86
S3_4	25.18	23.42	23.15	21.86	18.89
S4	26.65	26.64	26.15	23.53	17.95
